# The A736V TMPRSS6 polymorphism influences hepcidin and iron metabolism in chronic hemodialysis patients: TMPRSS6 and hepcidin in hemodialysis

**DOI:** 10.1186/1471-2369-14-48

**Published:** 2013-02-22

**Authors:** Serena Pelusi, Domenico Girelli, Raffaela Rametta, Natascia Campostrini, Carlo Alfieri, Michela Traglia, Paola Dongiovanni, Giovanna Como, Daniela Toniolo, Clara Camaschella, Piergiorgio Messa, Silvia Fargion, Luca Valenti

**Affiliations:** 1 Department of Pathophysiology and Transplantation, Internal Medicine, Università degli Studi di Milano, Fondazione IRCCS Ca’ Granda Ospedale Policlinico Milano, Milano, Italy; 2 Department of Medicine, Internal Medicine, Università di Verona, Verona, Italy; 3 Nephrology, Fondazione IRCCS Ca’ Granda Ospedale Policlinico, Milano, Italy; 4 San Raffaele Research Institute, Università Vita-Salute, Milano, Italy; 5 Institute of Molecular Genetics, CNR Pavia, Italy

**Keywords:** Anemia, Chronic kidney disease, Erythropoietin, Genetics, Inflammation, Iron, Hemodialysis, Hepcidin, Hfe gene, Matriptase-2, Tmprss6

## Abstract

**Background:**

Aim of this study was to evaluate whether the A736V TMPRSS6 polymorphism, a major genetic determinant of iron metabolism in healthy subjects, influences serum levels of hepcidin, the hormone regulating iron metabolism, and erythropoiesis in chronic hemodialysis (CHD).

**Methods:**

To this end, we considered 199 CHD patients from Northern Italy (157 with hepcidin evaluation), and 188 healthy controls without iron deficiency, matched for age and gender. Genetic polymorphisms were evaluated by allele specific polymerase chain reaction assays, and hepcidin quantified by mass spectrometry.

**Results:**

Serum hepcidin levels were not different between the whole CHD population and controls (median 7.1, interquartile range (IQR) 0.55-17.1 vs. 7.4, 4.5-17.9 nM, respectively), but were higher in the CHD subgroup after exclusion of subjects with relative iron deficiency (p = 0.04). In CHD patients, the A736V TMPRSS6 polymorphism influenced serum hepcidin levels in individuals positive for mutations in the HFE gene of hereditary hemochromatosis (p < 0.0001). In particular, the TMPRSS6 736 V variant was associated with higher hepcidin levels (p = 0.017). At multivariate analysis, HFE and A736V TMPRSS6 genotypes predicted serum hepcidin independently of ferritin and C reactive protein (p = 0.048). In patients without acute inflammation and overt iron deficiency (C reactive protein <1 mg/dl and ferritin >30 ng/ml; n = 86), hepcidin was associated with lower mean corpuscular volume (p = 0.002), suggesting that it contributed to iron-restricted erythropoiesis. In line with previous results, in patients without acute inflammation and severe iron deficiency the “high hepcidin” 736 V TMPRSS6 variant was associated with higher erythropoietin maintenance dose (p = 0.016), independently of subclinical inflammation (p = 0.02).

**Conclusions:**

The A736V TMPRSS6 genotype influences hepcidin levels, erythropoiesis, and anemia management in CHD patients. Evaluation of the effect of TMPRSS6 genotype on clinical outcomes in prospective studies in CHD may be useful to predict the outcomes of hepcidin manipulation, and to guide treatment personalization by optimizing anemia management.

## Background

Patients with end stage renal disease (ESRD) undergoing chronic hemodialysis (CHD) are commonly affected by anemia, which is related to erythropoietin (Epo) deficiency, blood losses, and chronic inflammation [1]. Treatment is based on erythropoiesis stimulating agents in association with intravenous (i.v.) iron formulations, but is of often difficult to achieve and maintain the desired hemoglobin (Hb) levels without incurring in side effects [2,3].

ESRD is characterized by major alterations in iron metabolism including low transferrin saturation (TS), resulting in reduced iron availability for the erythroblasts, and hyperferritinemia [2,4]. Upregulation of serum levels of hepcidin, the hepatic hormone regulating systemic iron metabolism, has been proposed to explain the alterations of iron metabolism of CHD patients and the resistance to anemia treatment [5,6]. Increased serum levels of hepcidin have indeed been reported in ESRD and CHD [2,5,7-11]. In response to increased iron stores, hepcidin inhibits intestinal iron absorption and iron recycling from monocytes by binding and inactivating the iron exporter Ferroportin-1. The consequent inhibition of iron export from duodenocytes and macrophages results in decreased TS, and increases serum ferritin as a result of iron entrapment into macrophages. Increased hepcidin in ESRD may result from reduced glomerular filtration, subclinical inflammation, as hepcidin is an acute phase reactant, and increased iron stores due to chronic supplementation. On the other hand, hepcidin is downregulated by anemia, hypoxia, and erythropoietin [12].

The upregulation of hepcidin transcription in response to iron is mediated by a mechanism depending on the interaction of various proteins including the hereditary hemochromatosis protein HFE, and matriptase-2 (TMPRSS6). We previously reported that in CHD patients common HFE mutations that alter hepatic iron sensing [13] were associated with lower hepcidin levels relatively to iron stores [6,14], achievement of target Hb levels for lower doses of iron, and with reduced mortality due to sepsis and cardiovascular disease, previously linked to more intense iron supplementation [15-18]. These initial results are in line with the hypothesis that inhibition of hepcidin in CHD may improve anemia control, and even survival in CHD patients [2,3,19,20].

The TMPRSS6 gene encodes for matriptase-2, a membrane-bound protease that decreases hepcidin transcription by cleaving hemojuvelin. Rare loss-of-function germline mutations of TMPRSS6 cause iron-refractory iron-deficiency anemia related to extremely high hepcidin levels, whereas the common rs855791 polymorphisms resulting in the p.A736V substitution is a major determinant of iron status in healthy subjects. Indeed, in the general population the p.736 V allele (henceforth 736 V) has been associated with lower serum iron, higher hepcidin [20,21], and decreased Hb [22-24], due to a less efficient inhibition of hepcidin transcription [21]. Furthermore, the p.A736V polymorphism has been shown to influence iron overload in hereditary hemochromatosis and nonalcoholic fatty liver disease [25,26]. However, it is not known whether the A736V variant influences iron metabolism during chronic inflammation and renal failure.

In the hypothesis that increased hepcidin is involved in the deregulation of iron metabolism and the anemia of CHD, the aim of this study were to evaluate whether the TMPRSS6 A736V polymorphism influences hepcidin levels and erythropoiesis parameters in CHD patients.

## Methods

### Subjects

We considered 199 CHD patients treated at the Fondazione IRCCS Ca’ Granda Ospedale Maggiore Policlinico from June 2006 to June 2011 [14]. Patients were dialyzed with synthetic biocompatible membranes and bicarbonate dialysate thrice in week (t.i.w.), and given i.v. recombinant human Epo (Eprex®) t.i.w., at a dose aimed to maintain hemoglobin (Hb) between 10.5 and 12 g/dl. Iron was administered i.v. as Fe^3+^-gluconate (Ferlixit®) when TS was less than 30% or ferritin <200 ng/ml, and suspended when ferritin was above 500 ng/ml [27]. Iron infusion was started once weekly and titrated according to requirements.

Baseline venous blood samples for complete blood count, iron parameters, and markers of inflammation (tested by standard methods) were collected in the morning before hemodialysis (the first weekly session) at standardized times after the last administration of therapies potentially altering iron status and hepcidin release: one week after the last dose of i.v. iron, and 3 days after the last dose of Epo (all these conditions were contemporarily satisfied for all patients). Aliquots of serum samples for hepcidin-25 and hepcidin-20 measurement, which were stored at −80°C until the analysis, were available in a subset of 157 and 99 patients, respectively [6]. Transferrin (TF) levels were only available from 2007, so that this variable could not be included in multivariate analyses.

We further selected a subgroup of 86 patients without inflammation or severe iron deficiency at baseline evaluation, arbitrarily defined on the basis of C reactive protein (CRP) levels <1 mg/dl and serum ferritin concentration >30 ng/ml, to avoid the confounding effect of acute inflammation and severe iron deficiency.

As reference population for hepcidin levels and for the prevalence of the genetic variants under study, we randomly selected 188 unrelated controls from the database of Val Borbera study [20]. Controls were unrelated subjects, with normal Hb (12-16 g/dl in females, 14-18 g/dl in males), ferritin (30-200 ng/ml in females, 40-300 ng/ml in males), and TS (16-45%), absence of homozygosity for the C282Y HFE mutation, and normal kidney function (estimated glomerular filtration rate according to simplified MDRD >60 ml/min), matched for age (± 5 years) and sex with CHD patients (for 11 patients, no match could be found).

Clinical, genetic and demographic features of subjects included in the study are shown in Table 1. Each patient gave written informed consent. The study was conducted according to the principles contained in the Declaration of Helsinki. The protocol was approved by the Institutional Review Board of the Fondazione IRCCS Ca’ Granda hospital of Milan.

**Table 1:** Demographic, and clinical features of 199 consecutive CHD patients from Northern Italy with available evaluation of C282Y and H63D HFE genotype (183 with A736V TMPRSS6 evaluation) and 188 healthy controls

	**All patients**	**Ferritin >30 ng/ml and CRP <1 mg/dl**	**Controls**
**N=**	**199**	**86**	**188**
Gender F	79 (40)	40 (47)	82 (44)
Age years	64.3±14 ^c^	63.8±14	60.0±17
BMI Kg/m^2^	22.5±5 ^c^	21.9±4 ^c^	26.2±4.2
Dialysis duration months	34 {13-82}	37 {16-87}	-
Kt/V	1.3±0.2	1.3±0.2	-
Creatinine mg/dl	9.9±2.5 ^c^	10.0±3 ^c^	0.8±0.2
Active smoke	47 (24)	17 (20)	23 (16)
Albumin g/100 ml	3.7±0.5	3.8±0.4	-
CRP mg/dl	0.83 {0.4-2.5} ^c^	0.40 {0.3-0.6} ^c^	0.1 {0-0.2}
Hb g/dl	10.8±1.2 ^c^	10.9±1.1 ^c^	14.7±1.1
MCV fl	96±8 ^c^	96±8 ^c^	92±4
Serum iron μg/dl	55±23 ^c^	58±19 ^c^	98±25
Transferrin mg/dl	190±37 ^c^	182±32 ^c^	240±34
TS%	24.7±10 ^c^	26.5±10 ^c^	29.0±7
Ferritin ng/ml	265 {155-411} ^c^	280 {204-445} ^c^	84 {55-128}
Epo IU/Kg/week	100 {59-180}	106 {69-179}	0
Fe i.v. mg/month	94 {0-185}	93 {0-142}	0
Hepcidin-20 ^a^ nM	0.55 {0.55-5.3} ^c^	1.39 {0.55-5.8}	0.87 {0.55-3.35}
Hepcidin-20 detectable	44 (45)	23 (51)	104 (55)
Hepcidin-25 ^b^ nM	7.1 {0.55-17.1}	6.8 {0.55-17.6}	7.4 {4.5-11.9}
Hepcidin-25 / ferritin ^b^	0.024 ^c^	0.021 ^c^	0.080
	{0.007-0.067}	{0.005-0.051}	{0.046-0.14}

():% values, {}: interquartile range, CRP: C reactive protein, Hb: hemoglobin, MCV: mean corpuscular volume, TS: transferrin saturation, Epo: erythropoietin, IU: international units, i.v.: intravenous, wt: wild-type, available in 99 CHD patients (45 with ferritin >30 ng/ml and CRP <1 mg/dl) ^b^ available in 157 CHD patients (57 with ferritin >30 ng/ml and CRP <1 mg/dl); ^c^ p < 0.05 vs. healthy controls.

### Genetic analysis and serum hepcidin assay

DNA was extracted from peripheral blood by the phenol-chloroform method. HFE genotype (C282Y and H63D variants) and the TMPRSS6 rs855791 C > T polymorphism, (p.A736V variant) were assessed by sequence allele specific PCR as previously described [25,28]. Random samples were confirmed by direct sequencing. Quality controls were performed to verify the reproducibility of the results. Valid genotypic data were obtained for 100% of subjects analyzed. For 13 patients (6.5%) only HFE genotype was available from a previous study, due to the lack of possibility to get the consent for new genetic studies.

For hepcidin measurement, we used a protocol based on SELDI-TOF mass spectrometry and copper-loaded immobilized metal-affinity capture ProteinChip arrays (IMAC30-Cu^2+^) [29], extensively validated in previous studies [13,20,21]. Concentrations of serum hepcidin-25 and hepcidin-20 were expressed as nM.

### Statistical analysis

Results are expressed as means ± SD for normally distributed variables and as median {interquartile range} for non-normally distributed variables. Variables were correlated by Spearman’s rho test, and data compared between groups by t-test or Wilcoxon test, according to data distribution. Frequencies were compared by Chi-square test. We also evaluated the hepcidin-25/ferritin ratio (H/F), an established marker of adequacy of hepcidin response to iron stores. Independent predictors of serum hepcidin-25 and Epo requirements were evaluated by multivariate analysis (generalized linear model, GLM) including the variables identified as significantly associated with hepcidin at univariate analysis and available for all subjects, as specified in the result section. Log transformations were applied to normalize skewed variables before multivariate analysis. Results were considered significant when p was lower than 0.05 (two-tailed).

## Results

### Frequency distribution of HFE and TMPRSS6 variants and hepcidin levels in patients and controls

The frequency distribution of the C282Y and H63D HFE variants and A736V of the TMPRSS6 variant did not violate Hardy-Weinberg equilibrium in both patients and controls (p > 0.1; Table 2), and was not significantly different between the two groups (p = ns). Serum hepcidin-25 levels were not significantly different between the whole group of CHD patients and controls (Table 1), whereas H/F ratio was lower in patients (Table 1). One hundred-four (52.3%) of CHD patients were classified as “iron-deficient” on the basis of guidelines for iron treatment in CHD, which takes into account the effect of inflammation on ferritin (ferritin <200 ng/ml and TS <30%) [27]. After the exclusion of these subjects, hepcidin-25 was higher in patients (9.31 {3.11-22.4} nM) than in controls (p = 0.04).

**Table 2:** C282Y and H63D HFE and A736V TMPRSS6 genotypes of 183 CHD patients and 188 healthy controls from the Val Borbera study (p = ns)

	**All patients**	**Controls**
HFE C282Y and H63D*	N = 199	N = 188
wt/wt	139 (69.0)	112 (59.6)
H63D/wt	46 (23.0)	54 (28.7)
H63D/H63D	5 (2.5)	8 (4.3)
C282Y/wt	8 (4.0)	12 (6.4)
C282Y/H63D	3 (1.5)	2 (1.0)
TMPRSS6 A736V	N = 183	N = 188
A/A	61 (33.3)	60 (31.9)
A/V	75 (41.0)	93 (49.5)
V/V	47 (25.7)	35 (18.6)

### Clinical determinants of hepcidin in CHD patients

Clinical variables associated with hepcidin-25 levels in CHD patients are shown in Table 3. Hepcidin-25 was correlated with ferritin and CRP levels, and negatively associated with TF. In patients without severe iron deficiency and with normal CRP levels (Table 4), hepcidin-25 was correlated with ferritin, and inversely correlated with TF and mean corpuscular volume (MCV) values.

**Table 3:** Clinical and genetic variables associated with hepcidin-25 levels at Spearman’s rho test (univariate analysis) and multivariate analysis in CHD patients from Northern Italy

	**Univariate**		**GLM model 1**		**GLM model 2**	
	**R**	**P**	**Estimate**	**p**	**Estimate**	**p**
Ferritin ng/ml	+0.30	0.0001	0.27	0.004	0.27	0.005
CRP mg/dl	+0.29	0.0004	0.29	0.01	0.28	0.010
Transferrin mg/dl	−0.20	0.03	-	-	-	-
Epo IU/Kg/week	+0.16	0.06	0.18	0.94	-	-
HDL mg/dl	−0.20	0.06	-	-	-	-
Albumin g/l	−0.15	0.06	-	-	-	-
TMPRSS6 A/A HFE muts + vs. other genotypes	−0.19	0.02	-	-	−0.17	0.048

Generalized linear model (GLM) 1 included clinical variables correlated with hepcidin-25 at univariate analysis (reported for p < 0.1), which were available in the majority of patients, i.e. ferritin, CRP levels, and Epo dose (157 subjects included). The second model considered the major clinical independent determinants of hepcidin, plus the genetic factors analyzed (143 subjects included).

CRP: C reactive protein; Epo: erythropoietin, IU: international units, HDL: high density lipoprotein cholesterol; HFE muts +: positive for HFE mutations; GLM: generalized linear model.

**Table 4:** **Clinical variables associated with hepcidin-25 levels at Spearman’s rho test (univariate analysis) in 86 CHD patients from Northern Italy with ferritin levels > 30 ng/ml and CRP < 1 mg/dl (the characteristics of this subset of patients are reported in Table**1**and related results; associated variables are shown for p < 0.1)**

	**R**	**p**
Ferritin ng/ml	+0.38	<0.0001
MCV fl	−0.47	0.002
Transferrin mg/dl	−0.23	0.012
TS%	+0.23	0.069
CRP mg/dl	+0.21	0.095

MCV: mean corpuscular volume; TS: transferrin saturation; CRP: C reactive protein.

The method used for the assessment of hepcidin-25 allows also the quantification of hepcidin-20, an amino-terminal truncated isoform, which is postulated to represent a degradation product of hepcidin-25 with no activity on iron metabolism, but possibly involved in antimicrobial response. Regarding the truncated hepcidin isoform (hepcidin-20) [30], the absolute levels were slightly lower in the whole CHD group than in controls, but these data were available in a limited subgroup of patients, and the prevalence of detectable hepcidin-20 levels was the same as in controls. On the other hand, absolute hepcidin-20 levels were slightly higher in the CHD subgroup of patients without functional iron deficiency (Table 1), while the difference did not reach statistical significance. Because of the small subgroups that could be analyzed, it is possible that these nominal differences represent false positive results, and no definite conclusion can be drawn. Variables associated with hepcidin-20 levels are shown in Table 5. The major determinant of hepcidin-20 was hepcidin-25, but active smoking was also independently associated with lower hepcidin-20. In control subjects, serum hepcidin-20 levels were not significantly lower in active smokers vs. non-smokers and previous smokers (median 0, IQR {2.99-6.38}, vs. 1.67 {3.68-6.59} nM, respectively; p = 0.17), even after correction for hepcidin-25 (p = 0.08).

**Table 5:** Clinical variables associated with hepcidin-20 levels at at Spearman’s rho test (univariate analysis) and multivariate generalized linear model (GLM) in 99 CHD patients from Northern Italy (reported for p < 0.1 for clinical variable)

	**Univariate**		**GLM**	
	**R**	**P**	**Estimate**	**p**
Hepcidin-25 nM	+0.60	<0.0001	+0.63	<0.0001
Active smoke	−0.25	<0.0001	−0.27	0.0016
Transferrin mg/dl	−0.23	0.032	-	-
TS%	+0.21	0.039	+0.13	0.22
TMPRSS6 A/A HFE muts + vs. other genotypes	−0.18	0.12	+0.13	0.99

TS: transferrin saturation; HFE muts +: positive for HFE mutations; GLM: generalized linear model.

### Effect of TMPRSS6 and HFE variants on hepcidin, iron, and erythropoiesis

**Figure 1:**
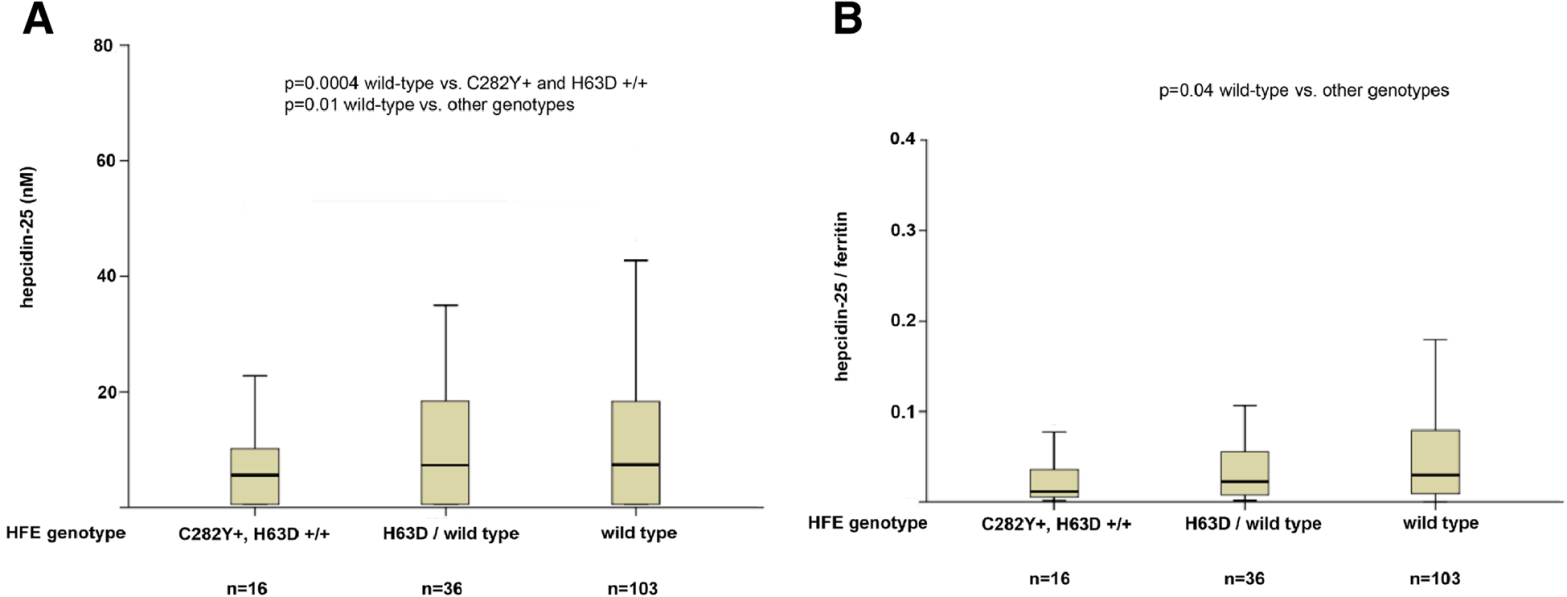
Effect of HFE C282Y and H63D genotype status (wild-type, heterozygosity for the H63D mutation, other genotypes) on hepcidin-25 levels (panel A), and hepcidin-25 / ferritin ratio (panel B) in 155 CHD patients from Northern Italy.

The effect of HFE genotype (wild-type, heterozygosity for C282Y and H63D mutations, and other genotypes) on hepcidin-25 in CHD patients is shown in Figure 1A. Hepcidin-25 was lower in patients with HFE mutations than in those without (p = 0.01), in particular in those carrying the C282Y mutation or homozygous for the H63D mutation (p = 0.0004). The effect of HFE genotype on the H/F ratio is shown in Figure 1B. The H/F ratio was lower in patients with HFE mutations than in those without (p = 0.04). Therefore, we grouped together patients with any HFE mutation in further analyses, in order to better characterize the effect of TMPRSS6 genotype.

**Figure 2:**
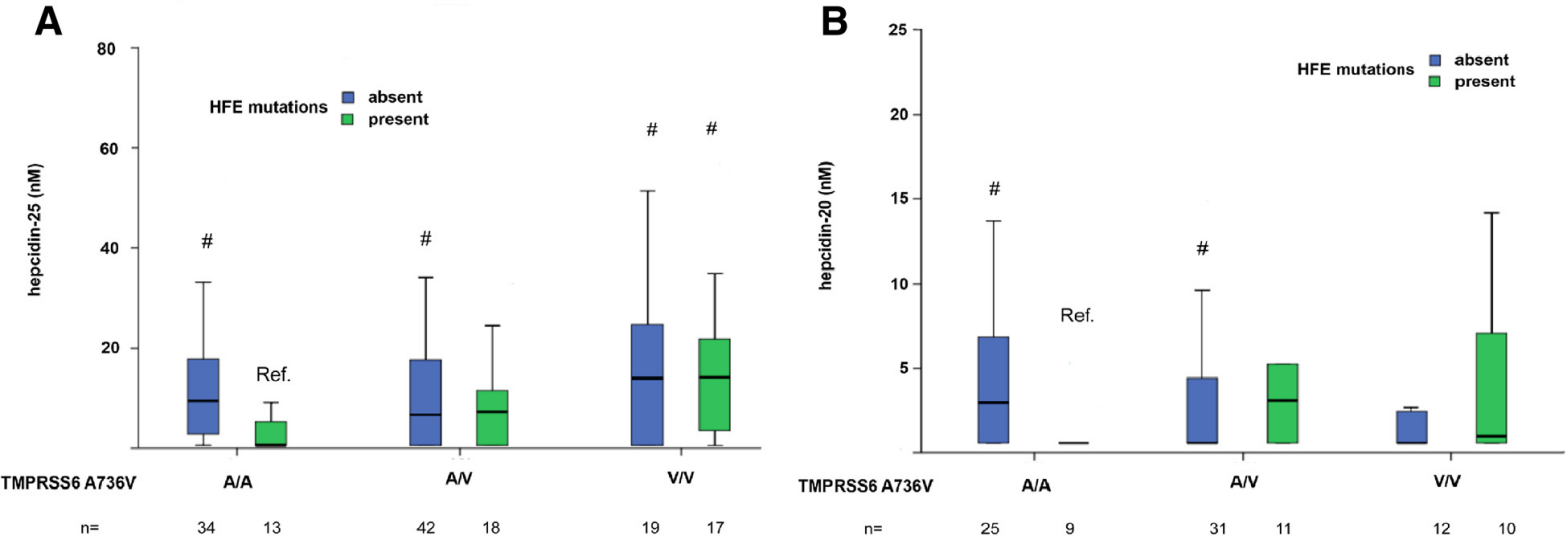
Combined effect of HFE C282Y and H63D and TMPRSS6 rs855791 (A736V) polymorphisms on hepcidin-25 levels in 143 (panel A), and on hepcidin-20 in 99 (panel B) CHD patients from Northern Italy. # p < 0.05 vs. TMPRSS6 A/A and HFE mutations present.

The combined HFE (presence or absence of HFE mutations) and TMPRSS6 A736V genotypes influenced serum hepcidin-25 levels (p < 0.0001; Figure 2A). In line with the hypothesized negative effect of HFE mutations and of the 736A allele on hepcidin transcription, patients negative for HFE mutations had higher hepcidin-25 levels than patients with 736A/A and positive for HFE mutations (p < 0.05). Furthermore, in patients with HFE mutations, those with the 736 V/V genotype had higher hepcidin-25 than those with the 736A/A genotype (p = 0.017). Similar results were obtained for the H/F ratio in CHD patients, i.e. the 736 V/V genotype was associated with significantly higher H/F than the 736A/A genotype in patients with, but not in those without HFE mutations (not shown).

At multivariate analysis (Table 3), the HFE positive 736A/A genetic status was associated with lower hepcidin-25 levels independently of ferritin and CRP levels (p = 0.048).

Patients negative for HFE mutations had higher hepcidin-20 levels than patients with 736A/A plus HFE mutations (Figure 2B). At multivariate analysis (Table 5), the effect of genetic factors on hepcidin-20 levels was not independent of hepcidin-25 levels.

Besides with hepcidin-25 values, as in the general population [20] the presence of HFE mutations was associated with lower TF levels (p = 0.03), and with a lower dose of iron supplementation to achieve the Hb target (p = 0.03).

TMPRSS6 A736V polymorphism was not associated with Hb levels and iron parameters in the overall CHD cohort. In order to avoid the confounding effect of acute inflammation and severe iron deficiency, we analyzed whether the TMPRSS6 A736V polymorphism influenced iron parameters and erythropoiesis in patients with CRP < 1 mg/dl and ferritin > 30 ng/ml. Results are presented in Table 6. In this subset of patients, the number of “high hepcidin” 736 V alleles was correlated with higher Epo requirement to control anemia (p = 0.027). In line with the negative effect of increased hepcidin on iron availability and erythropoiesis, there was also a trend for an association between the 736 V allele and lower iron and MCV values. At multivariate analysis adjusted for CRP levels, the number of 736 V TMPRSS6 alleles carried by CHD patients was associated with the weight-adjusted Epo dosage required to achieve the target Hb levels (p = 0.02, estimate coefficient 45, 95% c.i. 7-82).

**Table 6:** **Association of TMPRSS6 A736V polymorphism (736 V allele, additive model) with iron and erythropoietic parameters at Spearman’s rho test (univariate analysis) in 86 CHD patients from Northern Italy with ferritin > 30 ng/ml and CRP < 1 mg/dl (the characteristics of this subset of patients are reported in Table**1**and related results; associated variables are shown for p < 0.1)**

	**R**	**p**
Serum iron μg/ml	−0.26	0.077
CRP mg/dl	+0.22	0.061
MCV fl	−0.28	0.061
Epo IU/Kg/week	+0.28	0.027

CRP: C reactive protein; MCV: mean corpuscular volume; Epo: erythropoietin; IU: international units.

## Discussion

In CHD patients, it is usually difficult to control anemia because of a complex derangement of iron metabolism, which is due to chronic inflammation, blood losses, and concomitant Epo administration [2]. Increased serum levels of hepcidin, the hepatic hormone regulating iron metabolism, have been suggested to contribute to the functional iron deficiency that limit erythropoiesis in CHD [2,3,31]. Differently from what reported in smaller series of patients with unmatched controls, including previous studies from our group [5-8,32,33], we found that hepcidin-25 levels were not significantly increased in the whole CHD population. Nevertheless, accordingly to the previous reports [5-8,32,33], the major determinants of hepcidin were serum ferritin and CRP levels. The failure to confirm a relative hyper-hepcidinemia in the overall CHD cohort could be explained by a number of reasons: i) the inclusion of a large and less selected CHD population compared to previous studies, more closely reflecting patients observed in clinical practice, including those requiring high doses of Epo and with relative iron deficiency (factors both known to reduce hepcidin), as suggested by higher hepcidin levels in patients without functional iron deficiency; ii) the relatively low average iron stores in this cohort (as reflected by median serum ferritin levels of only 265 ng/ml, Table 1) because of a local policy aimed at minimizing iron supplementation, due to long standing interest in iron metabolism and the side effects of iron overload; and iii) at variance with previous studies, e.g. Zaritsky et al. [32], the meticulous matching of the controls for age and gender, recently established as major determinants of hepcidin-25 at population level [20,34], as well as the systematic exclusion of even subclinical iron deficiency in controls, which both contributed to a more realistic comparison of hepcidin-25 levels than those made until now. Accordingly with these considerations, however, when CHD patients with relative iron deficiency were excluded from these analyses, hepcidin-25 was actually higher in CHD than in controls. Anyway, the comparison of hepcidin levels between CHD patients and controls was not the main aim of the present study, which was not therefore specifically designed to achieve this goal. Our results in this sense need to be confirmed in similarly large patient populations and matched controls.

Clearance of hepcidin by hemodialysis [29] may possibly compensate for increased production, and explain the reduced H/F ratio in patients compared to controls. Notwithstanding, in patients without severe iron deficiency and active inflammation at the time of evaluation, hepcidin was associated with lower MCV, i.e. with iron-restricted erythropoiesis, suggesting that it negatively influences iron availability to the erythron, and that it represents a potential therapeutic target to improve anemia management.

The specific aim of this study was to evaluate whether the A736V TMPRSS6 polymorphism regulating hepcidin transcription, a determinant of iron-restricted erythropoiesis in the general population [21,35], influences hepcidin levels and erythropoiesis in CHD. To increase the power of this analysis, patients were stratified for the presence of loss-of-function HFE mutations, that we preliminarily confirmed to influence hepcidin in this series [6]. The major finding was that the 736 V TMPRSS6 loss-of-function variant appears to modulate the effect of HFE mutations on hepcidin. Indeed, the A736V polymorphism influenced serum hepcidin in patients positive for HFE mutations. This suggests that the 736 V variant with defective proteolytic activity determining increased hepcidin transcription [21] may abrogate the inhibitory effect of HFE mutations on hepcidin. Thus, the A736V TMPRSS6 variant appears as a modifier of the phenotypic expression of HFE mutations in patients with CHD, who are characterized by chronic subclinical inflammation.

Furthermore, in patients without overt iron deficiency and acute inflammation, the 736 V variant was associated with higher hepcidin levels and with higher requirement of Epo for anemia management, thus suggesting that the effect of TMPRSS6 genotype translates into clinically detectable differences in erythropoiesis. Importantly, at multivariate analysis the association between TMPRSS6 genotype and Epo maintenance dose was independent of subclinical inflammation, as indicated by CRP levels. These data are in line with the association between TMPRSS6 736 V with hepcidin levels, and in turn with the positive association of hepcidin with the Epo maintenance dose in the same subgroup. Therefore, inhibition of hepcidin might be helpful for a better control of anemia in patients predisposed to high hepcidin release [19]. Evaluation of the impact of HFE and TMPRSS6 genotype on the survival of CHD patients after adequate follow-up would be instrumental to fully define their clinical impact.

## Conclusions

In conclusion, in CHD patients the A736V TMPRSS6 genotype influences hepcidin levels, and in the absence of acute inflammation and severe iron deficiency, also erythropoiesis and anemia management. Evaluation of the effect of TMRPSS6 genotype on clinical outcomes in prospective studies in CHD patients may be useful to predict the outcomes of hepcidin manipulation, to develop new approaches to optimize anemia management, and to guide treatment personalization.

## Abbreviations

b.i.w: Bis in week; t.i.w: Thrice in week; CHD: Chronic hemodialysis; CRP: C reactive protein; Epo: Erythropoietin; ESRD: End stage renal disease; HFE: Hemochromatosis gene; H/F: Hepcidin/ferritin ratio; i.v: Intravenous; MCV: Mean corpuscular volume; TMPRSS6: Trans-membrane protease serine 6, or matriptase-2; TF: Transferrin; TS: Transferrin saturation.

## Competing interests

There is no conflict of interest relevant to this manuscript to disclose. *The work was supported by the following grants*: FIRST Università degli Studi di Milano 2007, 2008 (LV, SF: http://www.unimi.it); Ricerca corrente Ospedale Maggiore Policlinico 2006 and 2008 (LV, SF; http://www.policlinico.mi.it); and Centro per lo Studio delle Malattie del Fegato e del Metabolismo.

## Authors’ contributions

LV designed the study; SP and LV analyzed and interpreted the data, and wrote the manuscript draft; SP, RR, and PD performed genetic analyses and contributed to data analyses; NC evaluated serum hepcidin; NC, DG, CC, MT, and DT collected and analyzed the Val Borbera cohort; CA and GC collected the clinical data of patients; DG, CC, PM, SF, MT, DT contributed to data interpretation and to manuscript drafting. All Authors read and approved the final manuscript version.

## Authors’ information

Luca Valenti is assistant professor of Internal Medicine at Università degli Studi di Milano, Fondazione Ca’ Granda IRCCS Ospedale Maggiore Policlinico. He coordinates a research team contributing to this paper, and his main research interests include iron metabolism and metabolic liver diseases. He has an established collaboration with the group of P. Messa, head of the Nephrology Unit of the Fondazione IRCCS Ca’ Granda, and D. Girelli of the University of Verona, an international recognized expert in the field of hepcidin, for the study of the role of genetic factors in the regulation of iron metabolism in hemodialysis. C. Camaschella is one of the leading scientists in the iron metabolism field having contributed to elucidate the genetic basis of hereditary hemochromatosis and the secretary of the International Bioiron Society.

## Pre-publication history

The pre-publication history for this paper can be accessed here:

http://www.biomedcentral.com/1471-2369/14/48/prepub
